# Active inference, eye movements and oculomotor delays

**DOI:** 10.1186/1471-2202-14-S1-P133

**Published:** 2013-07-08

**Authors:** Laurent U Perrinet, Rick A Adams, Karl Friston

**Affiliations:** 1 The Wellcome Trust Centre for Neuroimaging, University College London, Queen Square, London WC1N 3BG, UK; 2 Institut de Neurosciences de la Timone, CNRS - Aix-Marseille Université, Marseille, France

## 

We consider the problem of sensorimotor delays in the optimal control of (smooth) eye movements under uncertainty. Specifically, we consider delays in the visuo-oculomotor loop and their implications for active inference. Active inference uses a generalisation of Kalman filtering to provide Bayes optimal estimates of hidden states and action in generalised coordinates of motion. Representing hidden states in generalised coordinates provides a simple way of compensating for both sensory and oculomotor delays. The efficacy of this scheme is illustrated using neuronal simulations of pursuit initiation responses, with and without compensation. We then consider an extension of the generative model to simulate smooth pursuit eye movements - in which the system believes both the target and its centre of gaze are attracted to a (fictive) point moving in the visual field. Finally, the generative model is equipped with a hierarchical structure, so that it can recognise and remember unseen (occluded) trajectories and emit anticipatory responses. These simulations speak to a straightforward and neurobiologically plausible solution to the generic problem of integrating information from different sources with different temporal delays and the particular difficulties encountered when a system - like the oculomotor system - tries to control its environment with delayed signals.

**Figure 1:**
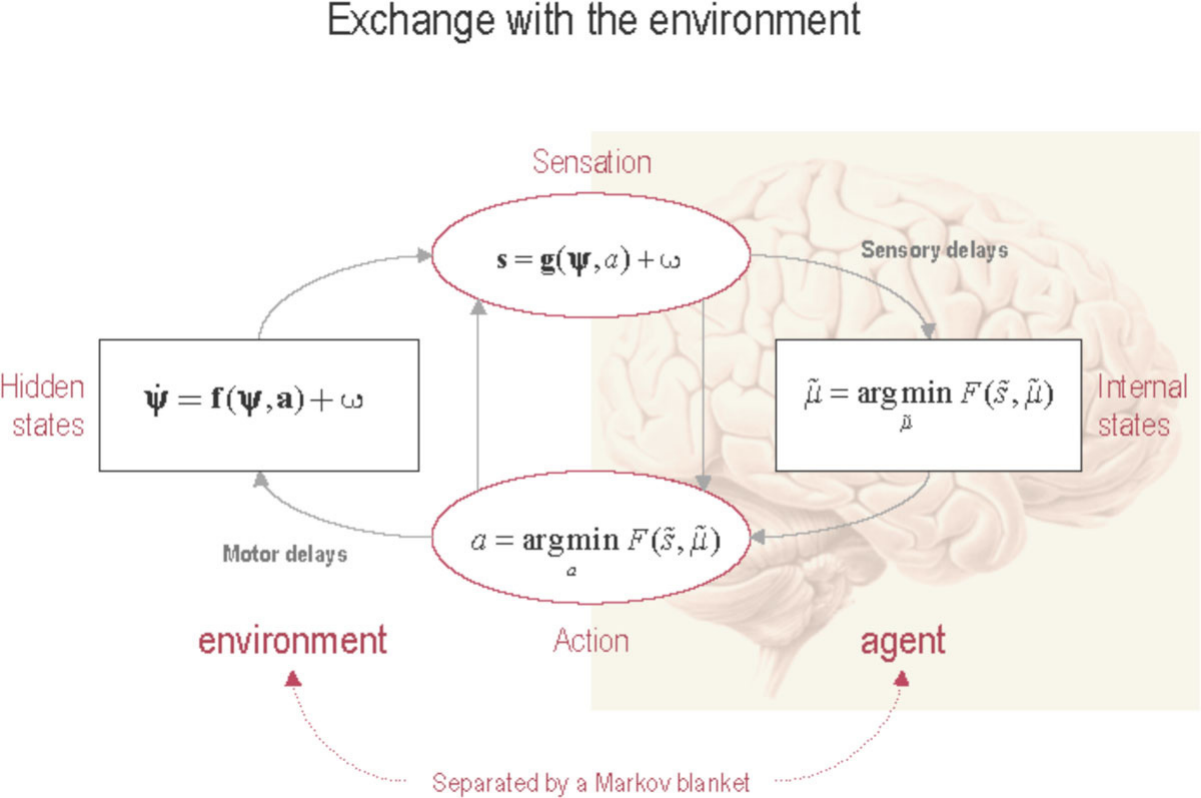
**This schematic shows the dependencies among various quantities modelling exchanges of an agent with the environment**. It shows the states of the environment and the system in terms of a probabilistic dependency graph, where connections denote directed dependencies. The quantities are described within the nodes of this graph - with exemplar forms for their dependencies on other variables (see main text). Hidden (external) and internal states of the agent are separated by action and sensory states. Both action and internal states - encoding a conditional probability density function over hidden states - minimise free energy. Note that hidden states in the real world and the form of their dynamics can be different from that assumed by the generative model; this is why hidden states are in bold. See main text for further details.

